# Unraveling the role of LINC02657 in clear cell renal cell carcinoma: insights into tumor aggression, immune modulation, and treatment response

**DOI:** 10.3389/fimmu.2026.1735169

**Published:** 2026-02-17

**Authors:** Juncheng Tong, Yang Liu, Aifa Tang, Xiangguo Xiong, Lifang Liu, Zhenqing Fan, Cheng Qian, Hongbing Mei, Han Wang

**Affiliations:** 1 Department of Urinary Surgery, Shenzhen Second People’s Hospital, the First Affiliated Hospital of Shenzhen University, Shenzhen, Guangdong, China; 2 Luohu Clinical College of Shantou University Medical College, Shantou University Medical College, Shantou, Guangdong, China; 3 Science and Educational Center, Luohu Hospital Group, The third Affiliated Hospital of Shenzhen University Shenzhen, Shenzhen, Guangdong, China; 4 Department of Oncology, Yantian District People’s Hospital, Shenzhen, Guangdong, China; 5 Department of Urinary Surgery, Lianping County People’s Hospital, Heyuan, Guangdong, China

**Keywords:** biomarkers, clear cell renal cell carcinoma, LINC02657, migration, proliferation

## Abstract

**Background:**

The role of long non-coding RNA LINC02657 in clear cell renal cell carcinoma (ccRCC) is poorly defined. This study aims to characterize its expression, clinical relevance, and oncogenic functions in ccRCC.

**Methods:**

We analyzed LINC02657 expression in pan-cancer and ccRCC cohorts from TCGA and ICGC. Prognostic value for overall (OS) and disease-specific survival (DSS) was evaluated using Kaplan-Meier and multivariate Cox regression. Functional mechanisms were investigated via Gene Set Enrichment Analysis (GSEA) and *in vitro* assays in 786-O and ACHN cells following LINC02657 knockdown, assessing proliferation, migration, invasion, and epithelial-mesenchymal transition (EMT). Drug sensitivity analysis was conducted using the GDSC2 database.

**Results:**

LINC02657 was significantly upregulated in ccRCC tissues. High LINC02657 expression predicted poorer OS and DSS and was an independent prognostic factor for adverse outcomes. GSEA linked it to cell cycle regulation and mitotic checkpoint signaling. Functional experiments demonstrated that LINC02657 knockdown effectively inhibited ccRCC cell proliferation, migration, and invasion, and promoted reversal of EMT toward an epithelial phenotype. Additionally, its expression was associated with immune cell infiltration and checkpoint molecule levels. Drug sensitivity profiling indicated that low LINC02657 expression enhanced sensitivity to chemotherapy agents, including docetaxel, gemcitabine, and 5-fluorouracil.

**Conclusions:**

LINC02657 is a critical oncogenic lncRNA in ccRCC, promoting tumor progression by regulating cell cycle, EMT, and immune microenvironment. It serves as a robust independent prognostic biomarker and a potential therapeutic target, offering valuable insights for personalized ccRCC treatment strategies.

## Introduction

Clear cell renal cell carcinoma (ccRCC) is the most common and clinically aggressive subtype of renal cell carcinoma (RCC), accounting for approximately 70–80% of all kidney cancer cases (1). Despite significant advancements in surgical techniques, targeted therapies, and immune checkpoint inhibitors, ccRCC remains a major cause of cancer-related mortality (2). The high incidence of metastasis and the tumor’s resistance to traditional treatments, such as chemotherapy and radiotherapy, emphasize the need for better prognostic markers and more effective therapeutic strategies (3). Increasingly, molecular investigations have provided insight into the genetic and epigenetic alterations that drive ccRCC pathogenesis (4, 5). However, many aspects of its molecular landscape remain incompletely understood, and new avenues for exploration are critical to improving clinical outcomes.

Long non-coding RNAs (lncRNAs) are a class of non-protein-coding RNA molecules that have been shown to regulate various cellular processes, including gene expression, chromatin modification, and post-transcriptional regulation (6). Over the past decade, lncRNAs have emerged as important regulators of tumor biology, influencing key aspects of cancer progression such as cell proliferation, migration, invasion, and metastasis. LncRNAs can modulate the tumor microenvironment, regulate immune responses, and impact drug sensitivity, making them attractive candidates for both diagnostic and therapeutic applications in oncology (7–10). In particular, dysregulated lncRNA expression has been implicated in the onset and progression of several cancers, including ccRCC (11, 12). While some lncRNAs have been well-studied and identified as crucial players in ccRCC, others remain underexplored and warrant further investigation.

LINC02657, a long non-coding RNA located on chromosome 10p15.1, has recently been identified as a potential candidate for further exploration in ccRCC, despite some preliminary findings suggesting its involvement in cancer progression, the exact molecular mechanisms by which LINC02657 influences ccRCC, its clinical relevance, and its potential as a therapeutic target have not been fully characterized (13, 14). Given the growing body of evidence linking lncRNAs to cancer biology, there is a compelling need to investigate the role of LINC02657 in ccRCC in greater detail. Understanding the molecular landscape of ccRCC, particularly in the context of non-coding RNA regulation, is crucial for identifying novel biomarkers and therapeutic targets. In this context, LINC02657 could potentially provide valuable insights into the molecular mechanisms driving ccRCC progression and may offer new opportunities for clinical management. The identification and validation of such biomarkers are key to the development of more personalized, targeted therapeutic approaches that could improve patient outcomes.

In our previous study (6), we systematically characterized molecular subtypes of clear cell renal cell carcinoma (ccRCC) based on programmed cell death (PCD)–related long non-coding RNAs and constructed a multigene prognostic risk model. In that work, LINC02657 was identified as one of seven PCD-related lncRNAs contributing to risk stratification and therapeutic response prediction at the population level. However, the biological function and mechanistic role of LINC02657 itself were not investigated. Building upon this prior hypothesis-generating analysis, the present study was designed as a gene-centric and mechanism-oriented extension, rather than a continuation of risk modeling. Here, we specifically selected LINC02657 for in-depth investigation to determine whether it represents a functionally active regulator of ccRCC progression, rather than merely a statistical component of a prognostic signature. Unlike our previous work, the current study does not construct or reuse a PCD-related risk model. Instead, we focus on elucidating the biological significance, functional effects, and potential mechanisms of LINC02657 in ccRCC. Through integrated bioinformatic analyses and experimental validation, we demonstrate that LINC02657 is aberrantly overexpressed in ccRCC, independently associated with unfavorable clinical outcomes, and functionally involved in tumor cell proliferation, migration, invasion, and epithelial–mesenchymal transition. Furthermore, we explore the relationship between LINC02657 expression and the tumor immune microenvironment, as well as its association with key oncogenic pathways, thereby providing mechanistic insights that extend beyond prognostic stratification alone. Collectively, this study advances LINC02657 from a risk model–associated lncRNA to a biologically and clinically relevant oncogenic driver, offering new perspectives that were not addressed by risk modeling approaches. Therefore, this study aims to systematically characterize the functional role and underlying mechanisms of LINC02657 in ccRCC progression.

## Materials and methods

### Data acquisition and processing

RNA-sequencing (RNA-seq) data and corresponding clinicopathological annotations for clear cell renal cell carcinoma (ccRCC) were retrieved from The Cancer Genome Atlas (TCGA) and the International Cancer Genome Consortium (ICGC) databases. Prior to analysis, samples with missing values or negligible gene expression were excluded to ensure data integrity. No additional filtering based on age, sex, tumor stage, grade, or treatment information was applied. TCGA-KIRC raw HTSeq-count data and ICGC normalized expression matrices were processed and analyzed separately to avoid cross-cohort batch effects. Raw count data from TCGA were used exclusively for differential expression analysis, while transcript expression values were transformed into transcripts per million (TPM) for downstream analyses, including survival analysis, immune infiltration estimation, and drug sensitivity prediction, to ensure comparability across samples. No direct merging or batch correction was performed between TCGA and ICGC datasets.

### Integrative bioinformatic analysis of LINC02657 expression and prognostic significance in ccRCC

Transcript abundance of LINC02657 was quantified in TPM values. Within each cohort, patients were stratified into high- and low-expression groups according to the median LINC02657 expression level. Survival outcomes—including Overall Survival (OS), Disease-Free Survival (DFS), Disease-Specific Survival (DSS), and Progression-Free Survival (PFS)—were evaluated using Kaplan–Meier analysis and log-rank tests. Univariate and multivariate Cox proportional hazards regression analyses were conducted to estimate hazard ratios (HRs) and 95% confidence intervals (CIs), adjusting for relevant clinicopathological covariates. Receiver operating characteristic (ROC) curve analysis was performed to assess the diagnostic performance of LINC02657. External consistency was assessed using the BSET database. The BSET platform is an online survival analysis tool integrating multiple public transcriptomic datasets and was used solely for auxiliary external validation.

### Bioinformatic and statistical analysis of LINC02657 in renal cell carcinoma

Differential gene expression analysis was performed using the DESeq2 package based on raw count data, with genes satisfying |log2 fold change| > 0.5 and FDR < 0.05 considered statistically significant. For downstream comparative analyses involving continuous variables, the Wilcoxon rank-sum test was applied. Survival analyses were conducted using the survival package, including Kaplan–Meier curves, log-rank tests, and Cox regression analyses. A prognostic nomogram was constructed using the rms package and validated by bootstrap resampling. Model performance was evaluated using the concordance index (C-index). All bioinformatic analyses were performed in R (version 4.2.2). Key R packages included DESeq2 (v1.38.0), limma (v3.54.0), clusterProfiler (v4.6.0), GSVA (v1.46.0), survival (v3.5-5), rms (v6.7-0), and estimate (v1.0.13).

### Differential expression and functional enrichment analysis of LINC02657-associated genes

Differentially expressed genes between high- and low-LINC02657 expression groups were identified based on DESeq2 results. Gene Set Enrichment Analysis (GSEA) was performed using the clusterProfiler package based on ranked gene lists derived from log2 fold change values. Gene sets with a normalized enrichment score (NES) |NES| > 1, nominal P value < 0.05, and false discovery rate (FDR) < 0.25 were considered statistically significant, in accordance with recommended GSEA guidelines. Gene Ontology (GO) and KEGG pathway enrichment analyses were conducted using over-representation analysis with multiple testing correction applied. Enrichment results were visualized using bar plots.

### Tumor microenvironment scoring and immune infiltration correlation analysis

The ESTIMATE algorithm was applied to bulk RNA-seq data to infer stromal and immune scores. Immune cell composition was estimated using the CIBERSORT algorithm with the LM22 signature matrix. Prior to deconvolution, gene expression data were used in their original normalized form as provided by TCGA, and no additional batch correction was applied, as all samples were derived from the same TCGA-KIRC cohort. Quantile normalization was disabled in CIBERSORT, in accordance with recommended practices for RNA-seq data. Only samples with CIBERSORT deconvolution P values < 0.05 were retained for downstream analysis. Spearman correlation analysis was performed to assess associations between LINC02657 expression and immune cell infiltration levels. These analyses were conducted for exploratory purposes and do not imply causal relationships.

### Drug sensitivity prediction based on LINC02657 expression

Associations between LINC02657 expression and drug sensitivity were explored using transcriptomic data and drug response profiles from the GDSC2 database. Analyses were conducted in an exploratory manner to identify potential correlations between gene expression and estimated drug response, without inferring clinical efficacy or therapeutic causality.

### Cell line cultures and LINC02657 knockdown

Human cell lines, including HK-2 (human normal renal tubular epithelial cells), 786-O, 769-P, ACHN, and CAKI-1 (renal cancer cell lines), were cultured in RPMI-1640 medium supplemented with 10% fetal bovine serum (FBS) and 1% penicillin-streptomycin, under standard conditions (37°C, 5% CO_2_). Cells were routinely passaged upon reaching 80-90% confluence. The RNA expression levels of LINC02657 in each cell line were assessed by quantitative reverse transcription polymerase chain reaction (qRT-PCR). In addition, LINC02657 expression was simultaneously examined in 30 paired renal cell carcinoma tissues and adjacent non-tumorous tissues collected from Shenzhen Second People’s Hospital. For LINC02657 knockdown, chemically synthesized small interfering RNAs (siRNAs) specifically targeting human LINC02657 were transfected into ACHN and 786-O cells. Plasmid-based transfection was performed using specific siRNA constructs targeting LINC02657. Transfection was carried out using Lipofectamine 3000 reagent (Thermo Fisher Scientific), following the manufacturer’s protocol. Three independent siRNA sequences targeting distinct regions of the LINC02657 transcript were designed and tested to minimize potential off-target effects, and the most efficient siRNA was selected for subsequent functional assays. The sequences of all siRNAs are provided in **Supplementary Table S1**. The efficiency of LINC02657 knockdown was assessed 48 hours post-transfection by quantitative real-time PCR (qRT-PCR). Total RNA was isolated using TRIzol reagent (Invitrogen), and cDNA synthesis was performed using the PrimeScript™ RT reagent Kit (Takara Bio).

### Cell proliferation assay

Cell proliferation was assessed using the Cell Counting Kit-8 (CCK-8) assay in 786-O and ACHN renal cancer cell lines following LINC02657 knockdown. Cells were seeded into 96-well plates at a density of 5 × 10^3 cells per well and incubated for 24 hours. The experimental group was subjected to LINC02657 knockdown via plasmid transfection, while the control group was transfected with a scramble vector. Cell proliferation was evaluated at 0h, 24h, 48h, and 72 hours post-transfection. At each time point, 10 µL of CCK-8 reagent (Dojindo Molecular Technologies, Japan) was added to each well, and the cells were incubated for an additional 2 hours at 37°C. The absorbance at 450 nm was measured, and the relative cell proliferation was calculated by normalizing the absorbance of the experimental group to that of the control group at each time point.

### EdU incorporation assay

To assess DNA synthesis, 786-O and ACHN cells were plated in 24-well plates and transfected with si-LINC02657 or negative control siRNA. After 48 h, EdU labeling was performed following the protocol provided with the EdU detection kit. Cells were then fixed and processed for fluorescent staining, and nuclei were counterstained with DAPI. Fluorescence images were captured using a fluorescence microscope, and EdU-positive cells were quantified from multiple randomly selected fields.

### Colony formation assay

After siRNA transfection, 786-O and ACHN cells were collected and seeded into 6-well plates at low density. Cells were maintained under standard culture conditions until discrete colonies became visible. Colonies were subsequently fixed and stained with crystal violet. Colonies consisting of more than 50 cells were counted, and the results were recorded for each group.

### Cell cycle analysis

Cell cycle distribution was examined by flow cytometry. Briefly, 786-O and ACHN cells were harvested 48 h after transfection with si-LINC02657 or si-NC. Cells were washed with PBS and fixed in 70% ethanol at 4°C. After fixation, cells were treated with RNase A and stained with propidium iodide. Samples were analyzed using a flow cytometer, and the proportions of cells in different cell cycle phases were calculated.

### Transwell migration and invasion assays

Cell migration and invasion were assessed using Transwell assays in ACHN and 786-O cell lines following LINC02657 knockdown. For migration, 2 × 10^4 cells were seeded in the upper chamber of a 24-well Transwell insert (8 μm pore size), with the lower chamber containing complete medium with 10% FBS as a chemoattractant. After 24 hours, non-migrated cells were removed, and migrated cells on the lower surface were fixed, stained with crystal violet, and counted under a microscope. For invasion, the chambers were pre-coated with Matrigel, and the procedure was performed similarly to the migration assay. After 48 hours, invading cells were stained and quantified.

### Western blot analysis of EMT markers

To assess the impact of LINC02657 knockdown on EMT-related proteins, Western blotting was performed in ACHN and 786-O cell lines. Cells were lysed in RIPA buffer (Beyotime, China) containing protease inhibitors (Roche, Switzerland), and protein concentrations were measured using a BCA assay (Beyotime, China). Equal protein amounts (30 µg) were separated by SDS-PAGE, transferred to PVDF membranes (Millipore, USA), and blocked with 5% non-fat milk. Membranes were incubated overnight at 4°C with primary antibodies against E-cadherin, N-cadherin, vimentin, and Slug (Cell Signaling Technology, USA; Abcam, UK). After incubation with HRP-conjugated secondary antibodies (Cell Signaling Technology, USA), protein bands were detected using ECL (Thermo Fisher Scientific, USA). GAPDH was used as the internal control. The following primary antibodies were used: anti-E-cadherin (Cell Signaling Technology, #3195, rabbit monoclonal antibody, 1:1000), anti-N-cadherin (Cell Signaling Technology, #13116, rabbit monoclonal antibody, 1:1000), anti-vimentin (Cell Signaling Technology, #5741, rabbit monoclonal antibody, 1:1000), anti-Slug (Cell Signaling Technology, #9585, C19G7 rabbit monoclonal antibody, 1:1000), and anti-GAPDH (Abcam, EPR16891, rabbit monoclonal antibody, 1:5000).

### Statistical analysis

All statistical analyses were performed using R software (version 4.2.2) and GraphPad Prism (version 9.0). Data are presented as mean ± standard deviation (SD). Comparisons between two groups were conducted using two-tailed Student’s t-tests, and multiple-group comparisons were analyzed by one-way ANOVA. Survival differences were evaluated using Kaplan–Meier analysis with log-rank tests, and Cox proportional hazards regression was applied to estimate hazard ratios (HRs) with 95% confidence intervals (CIs). Correlation analyses were performed using Spearman’s rank correlation. All *in vitro* experiments were independently repeated at least three times. A P value < 0.05 was considered statistically significant.

## Results

### Expression landscape of LINC02657 and its association with clinicopathological features in ccRCC

**Figure 1:**
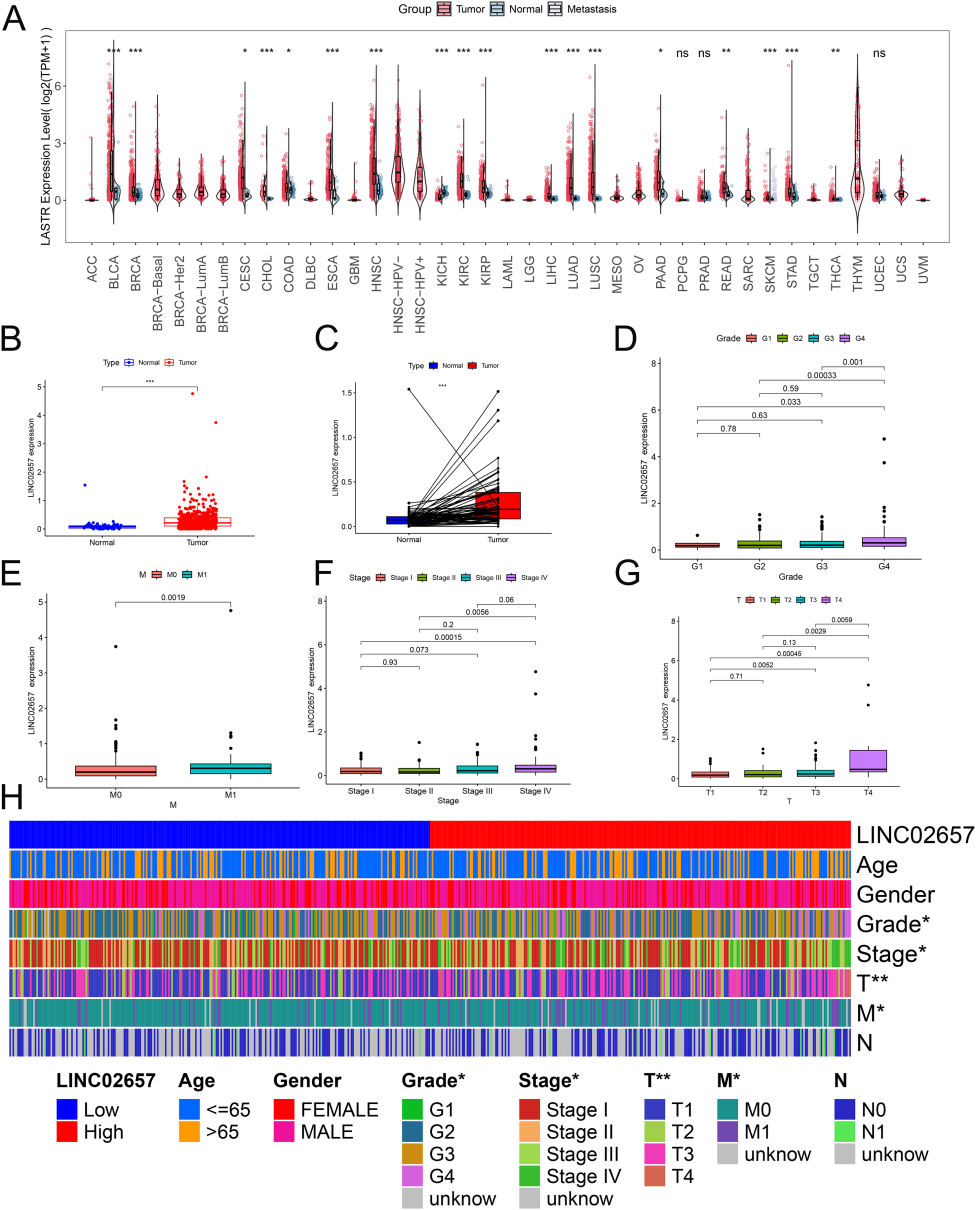
Expression profile of LINC02657 across malignancies and its association with clinicopathological features in clear cell renal cell carcinoma (ccRCC). **(A)** Pan-cancer differential expression analysis of LINC02657 across tumor and adjacent normal tissues using TCGA datasets. **(B)** Boxplot depicting LINC02657 expression levels in ccRCC tumor tissues versus matched normal renal tissues in the TCGA-KIRC cohort (***P < 0.001). **(C)** Paired dot plot analysis of LINC02657 expression in matched tumor and adjacent normal tissues from ccRCC patients (***P < 0.001). **(D–G)** Association between LINC02657 expression levels and key clinicopathological features, including tumor grade **(D)**, M stage **(E)**, clinical stage **(F)**, and T stage **(G, H)** Heatmap of LINC02657 expression across clinicopathological features in ccRCC. Columns represent individual patient samples and rows indicate clinical characteristics * indicates P < 0.05 and ** indicates P < 0.01.

A comprehensive pan-cancer analysis was initially conducted to characterize the expression profile of LINC02657 across malignancies. As illustrated in **Figure 1A**, LINC02657 was markedly upregulated in tumor tissues compared to adjacent non-tumor tissues in multiple cancer types, including bladder urothelial carcinoma(BC), breast invasive carcinoma(BRCA), cervical squamous cell carcinoma and endocervical adenocarcinoma(CESC), cholangiocarcinoma(CHOL), colon adenocarcinoma(COAD), esophageal carcinoma(ESCA), head and neck squamous cell carcinoma(HNSC), Kidney renal clear cell carcinoma (KIRC), Kidney renal papillary cell carcinoma (KIRP), Liver hepatocellular carcinoma (LIHC), lung adenocarcinoma(LUAD), lung squamous cell carcinoma(LUSC), prostate adenocarcinoma(PRAD), rectal adenocarcinoma(READ), skin cutaneous melanoma(SKCM), stomach adenocarcinoma(STAD), and thyroid carcinoma(THCA). Notably, this upregulation was not observed in Kidney Chromophobe (KICH), indicating potential tumor-specific regulation. Focusing specifically on ccRCC, LINC02657 was significantly overexpressed in tumor tissues relative to matched normal renal tissues, as demonstrated in **Figures 1B, C** (P < 0.001). To elucidate the clinical relevance of LINC02657 expression, its association with key clinicopathological parameters was systematically evaluated. As shown in **Figures 1D–G** and summarized in **Table 1**, high LINC02657 expression was significantly correlated with advanced tumor grade (P = 0.0434), clinical stage (P = 0.0175), primary tumor size (T stage, P = 0.0048) and distant metastasis (M stage, P = 0.0125). A heatmap representation in **Figure 1H** further illustrated the distribution pattern of LINC02657 expression across diverse clinical subgroups, highlighting its potential role as a biomarker of aggressive disease phenotypes in ccRCC.

**Table 1:** Correlation between LINC02657 expression and clinicopathological characteristics of bladder cancer patients.

Variable	Group	Total	Low	High	P value
Age	<=65	348(65.66%)	176(66.42%)	172(64.91%)	0.7838
	>65	182(34.34%)	89(33.58%)	93(35.09%)	
Gender	FEMALE	186(35.09%)	94(35.47%)	92(34.72%)	0.9275
	MALE	344(64.91%)	171(64.53%)	173(65.28%)	
Grade	G1	14(2.68%)	8(3.09%)	6(2.28%)	0.0434
	G2	227(43.49%)	120(46.33%)	107(40.68%)	
	G3	206(39.46%)	105(40.54%)	101(38.4%)	
	G4	75(14.37%)	26(10.04%)	49(18.63%)	
Stage	Stage I	265(50.28%)	146(55.3%)	119(45.25%)	0.0175
	Stage II	57(10.82%)	30(11.36%)	27(10.27%)	
	Stage III	123(23.34%)	59(22.35%)	64(24.33%)	
	Stage IV	82(15.56%)	29(10.98%)	53(20.15%)	
T	T1	271(51.13%)	150(56.6%)	121(45.66%)	0.0048
	T2	69(13.02%)	35(13.21%)	34(12.83%)	
	T3	179(33.77%)	79(29.81%)	100(37.74%)	
	T4	11(2.08%)	1(0.38%)	10(3.77%)	
M	M0	420(84.34%)	224(88.54%)	196(80%)	0.0125
	M1	78(15.66%)	29(11.46%)	49(20%)	
N	N0	239(93.73%)	116(95.08%)	123(92.48%)	0.5505
	N1	16(6.27%)	6(4.92%)	10(7.52%)	

### Prognostic and diagnostic significance of LINC02657 in renal cell carcinoma

**Figure 2:**
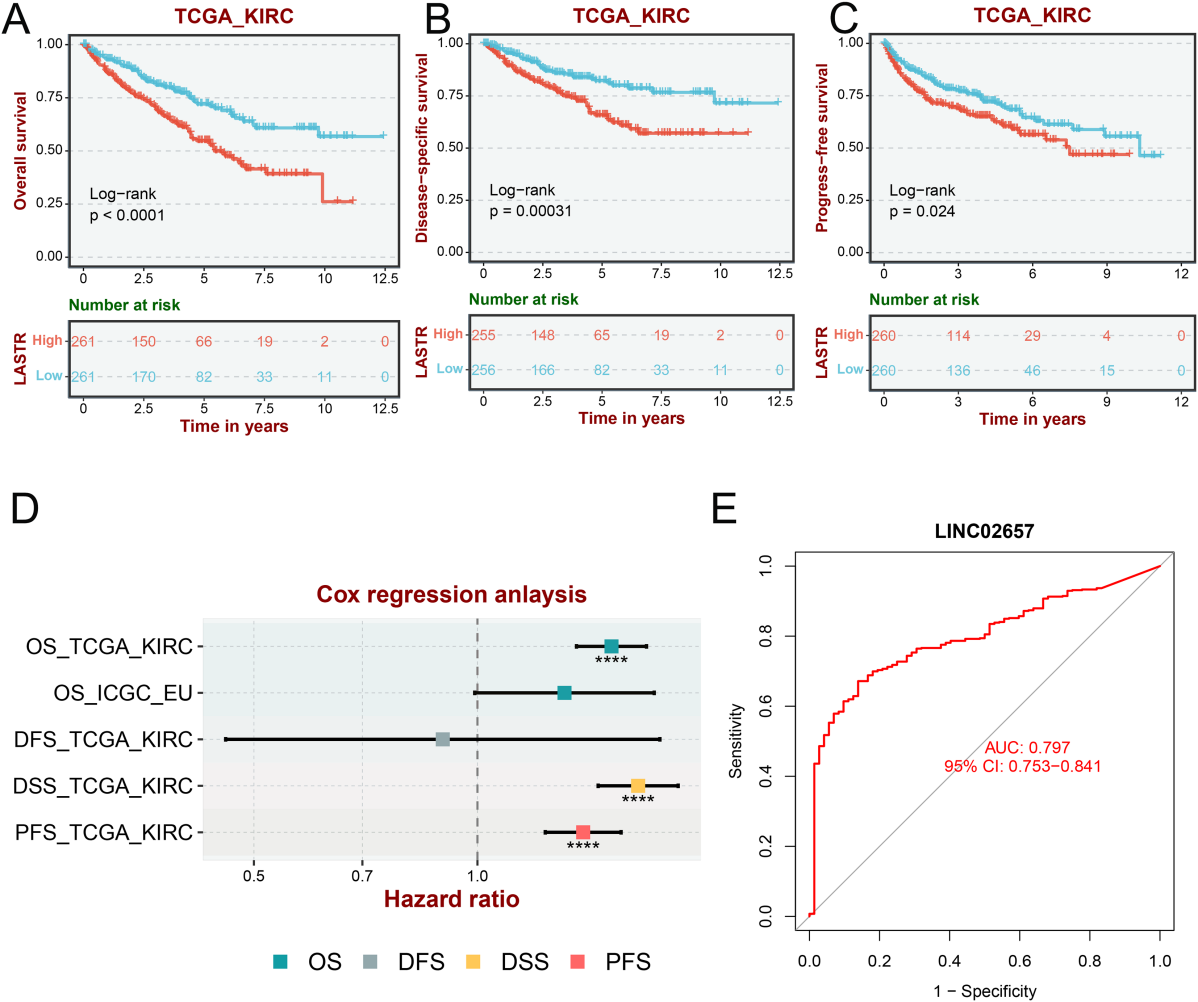
Prognostic and diagnostic value of LINC02657 in ccRCC. **(A–C)** Kaplan–Meier curves for Overall Survival (OS), Disease-Specific Survival (DSS), and Progression-Free Survival (PFS) stratified by LINC02657 expression in the TCGA-KIRC cohort (log-rank test). **(D)** Multivariate Cox regression forest plot showing hazard ratios (HRs) and 95% confidence intervals (CIs) for OS-TCGA, DSS-TCGA, and PFS-TCGA, OS-ICGC and DFS-TCGA. **(E)** ROC curve assessing the diagnostic performance of LINC02657 expression in ccRCC.

Kaplan-Meier survival analyses revealed that elevated LINC02657 expression was significantly associated with adverse clinical outcomes in the TCGA-KIRC cohort. Patients with high LINC02657 expression exhibited markedly reduced Overall Survival (OS) (log-rank P < 0.001), Disease-Specific Survival (DSS) (log-rank P = 0.00031), and Progression-Free Survival (PFS) (log-rank P = 0.024) compared to their low-expression counterparts (**Figures 2A-C**). These findings were robustly validated using the BSET database, demonstrating consistent prognostic trends across independent datasets. Multivariate Cox regression analysis further substantiated the prognostic relevance of LINC02657. As visualized in the forest plot (**Figure 2D**), high LINC02657 expression was associated with significantly elevated hazard ratios (HRs) for OS-TCGA, DSS-TCGA, and PFS-TCGA, with all HR estimates positioned to the right of the null line (HR = 1), indicating a higher risk of adverse outcomes. In contrast, no statistically significant associations were observed for OS-ICGC or DFS-TCGA (**Supplementary Figures S1A, B**), suggesting a context-dependent prognostic effect of LINC02657 that may vary with cohort-specific factors. Diagnostic performance was evaluated via ROC curve analysis (**Figure 2E**), which demonstrated that LINC02657 possesses substantial discriminatory power, yielding an AUC of 0.797 (95% CI: 0.753–0.841). Further supporting its prognostic value, time-dependent receiver operating characteristic (ROC) analysis demonstrated that LINC02657 expression could predict patient survival with area under the curve (AUC) values of 0.606, 0.637, and 0.654 for 1-year, 3-year, and 5-year survival, respectively (**Supplementary Figure S1C**). Notably, the expression level of LINC02657 was not associated with patient age or gender (**Supplementary Figures S1D, E**), suggesting its prognostic role is independent of these basic clinical parameters. This finding underscores the potential of LINC02657 as a biomarker capable of differentiating ccRCC patients with high precision.

### Prognostic significance of LINC02657 in renal cell carcinoma survival outcomes

**Figure 3:**
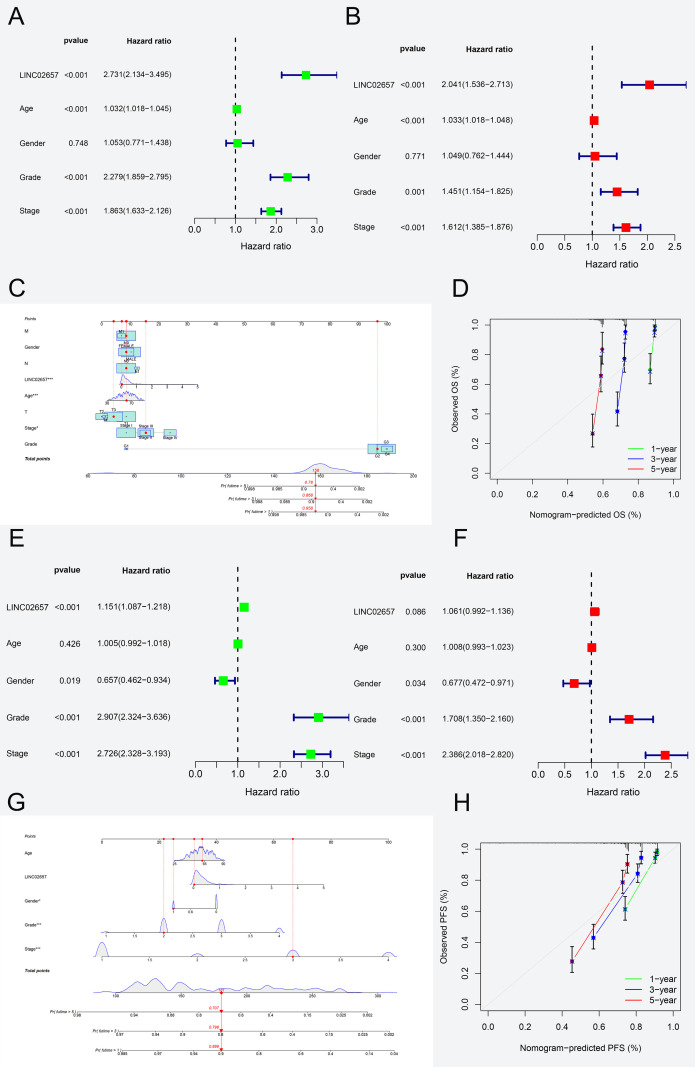
Prognostic modeling of LINC02657 expression in ccRCC survival outcomes. **(A, B)** Univariate **(A)** and multivariate **(B)** Cox regression analyses for Overall Survival in the TCGA-KIRC cohort, showing hazard ratios (HRs) and 95% confidence intervals (CIs) for clinicopathological variables and LINC02657 expression. **(C)** Nomogram integrating LINC02657 expression, tumor grade, stage, and age for individualized OS prediction. **(D)** Calibration curve assessing nomogram predictive accuracy. **(E, F)** Univariate **(E)** and multivariate **(F)** Cox regression analyses for Progression-Free Survival. **(G)** PFS nomogram and **(H)** corresponding calibration curve demonstrating robust model calibration.

In the analysis of overall survival (OS),univariate Cox regression (**Figure 3A**, **Table 2**) revealed significant associations between adverse prognosis and age (HR = 1.032, 95% CI: 1.018-1.045, P < 0.001), tumor grade (HR = 2.279, 95% CI: 1.859-2.795, P < 0.001), tumor stage (HR = 1.863, 95% CI: 1.633-2.126, P < 0.001), and high LINC02657 expression (HR = 2.731, 95% CI: 2.134-3.495, P < 0.001). Multivariate Cox regression (**Figure 3B**), adjusting for confounding factors, confirmed high LINC02657 expression as an independent prognostic factor for OS (HR = 2.041, 95% CI: 1.536-2.713, P < 0.001), with predictive strength surpassed only by tumor stage (HR = 1.612, 95% CI: 1.385-1.876, P < 0.001) and superior to tumor grade (HR = 1.451, 95% CI: 1.154-1.825, P = 0.001). The nomogram constructed from these variables (**Figure 3C**) demonstrated robust predictive performance, with calibration curves (**Figure 3D**) indicating high concordance between predicted and observed survival probabilities. For progression-free survival (PFS), univariate analysis (**Table 3**, **Figure 3E**) identified significant associations with gender (HR = 0.657, 95% CI: 0.462-0.934, P = 0.019), tumor grade (HR = 2.907, 95% CI: 2.324-3.636, P < 0.001), tumor stage (HR = 2.726, 95% CI: 2.328-3.193, P < 0.001), and high LINC02657 expression (HR = 1.151, 95% CI: 1.087-1.218, P < 0.001). However, multivariate analysis (**Figure 3F**) revealed attenuated prognostic significance for LINC02657 (HR = 1.061, 95% CI: 0.992–1.136, P = 0.086), while tumor grade (HR = 1.708, 95% CI: 1.350-2.160, P < 0.001) and stage (HR = 2.386, 95% CI: 2.018–2.820, P < 0.001) retained independent predictive value. The corresponding nomogram (**Figure 3G**) and calibration curve (**Figure 3H**) exhibited strong predictive accuracy. These findings underscore LINC02657 as a potent independent prognostic factor for OS in KIRC, with its predictive utility for PFS potentially modulated by traditional pathological factors such as tumor grade and stage, suggesting a more pronounced role in later stages of tumor progression.

**Table 2:** Univariate and multivariate analyses for overall survival.

Clinicopathological variables	Univariate			Multivariate		
	RR	95.0% CI	P-value	RR	95.0% CI	P-value
Age	1.032	(1.018−1.045)	<0.001	1.033	(1.018−1.048)	<0.001
Gender	1.053	(0.771−1.438)	0.748	1.049	(0.762−1.444)	0.771
Grade	2.279	(1.859−2.795)	<0.001	1.451	(1.154−1.825)	0.001
Stage	1.863	(1.633−2.126)	<0.001	1.612	(1.385−1.876)	<0.001
LINC02657	2.731	(2.134-3.495)	<0.001	2.041	(1.536-2.713)	<0.001

**Table 3:** Univariate and multivariate analyses for progression free survival.

Clinicopathological variables	Univariate			Multivariate		
	RR	95.0% CI	P-value	RR	95.0% CI	P-value
Age	1.005	(0.992−1.018)	0.426	1.008	(0.993−1.023)	0.300
Gender	0.657	(0.462−0.934)	0.019	0.677	(0.472−0.971)	0.034
Grade	2.907	(2.324−3.636)	<0.001	1.708	(1.350−2.160)	<0.001
Stage	2.726	(2.328−3.193)	<0.001	2.386	(2.018−2.820)	<0.001
LINC02657	1.151	(1.087−1.218)	<0.001	1.061	(0.992−1.136)	0.086

### LINC02657-associated differential gene expression and functional enrichment analysis

**Figure 4:**
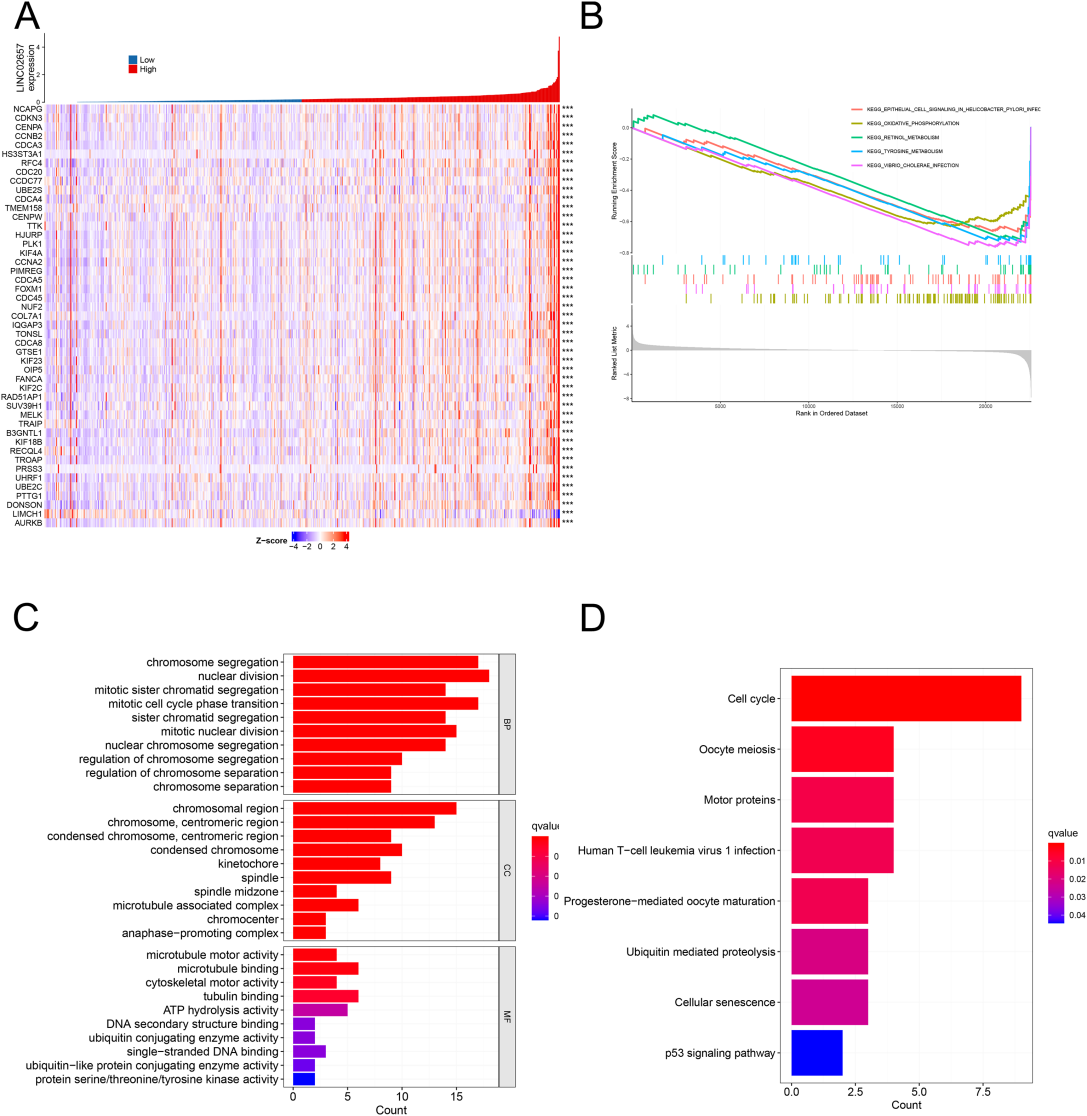
Differential gene expression and functional enrichment analyses associated with LINC02657 in ccRCC. **(A)** Heatmap depicting the top 50 differentially expressed genes (DEGs) between high and low LINC02657 expression groups in the TCGA-KIRC cohort. DEGs were identified using DESeq2, with |log_2_ fold change| > 0.5 and false discovery rate (FDR) < 0.05 considered statistically significant. **(B)** Gene Set Enrichment Analysis (GSEA) highlighting pathways enriched in LINC02657-associated DEGs, based on ranked gene lists derived from log_2_ fold change values. **(C)** Gene Ontology (GO) enrichment analysis summarizing the top biological processes, molecular functions, and cellular components associated with LINC02657 expression. **(D)** Kyoto Encyclopedia of Genes and Genomes (KEGG) pathway enrichment analysis revealing key pathways dysregulated in the context of LINC02657 overexpression. For enrichment analyses, adjusted P values (FDR < 0.05) were used to determine statistical significance. *** indicates P < 0.001.

As shown in **Figure 4A**, heatmap analysis of the top 50 differentially expressed genes, stratified by the median expression level of LINC02657, revealed distinct transcriptomic profiles between high and low expression groups. Gene Set Enrichment Analysis (GSEA) (**Figure 4B**) indicated significant enrichment in pathways associated with epithelial cell signaling in Helicobacter pylori infection, oxidative phosphorylation, retinol metabolism, tyrosine metabolism, and Vibrio cholerae infection, suggesting involvement in metabolic reprogramming and infection-related signaling. Gene Ontology (GO) enrichment analysis (**Figure 4C**) demonstrated that differentially expressed genes were primarily enriched in biological processes related to chromosome segregation, mitotic nuclear division, sister chromatid segregation, and cell cycle phase transition, while molecular function analysis highlighted significant enrichment in microtubule motor activity, ATPase activity, ubiquitin-conjugating enzyme activity, and DNA binding. Cellular component annotations revealed preferential localization to chromosomal regions, spindle apparatus, kinetochore, and the anaphase-promoting complex, implicating these genes in mitotic regulation and genomic integrity. KEGG pathway analysis (**Figure 4D**) further identified enrichment in key oncogenic pathways, including cellular senescence, ubiquitin-mediated proteolysis, oocyte meiosis, cell cycle progression, and the p53 signaling pathway, collectively indicating that LINC02657-associated gene dysregulation may contribute to tumorigenesis through aberrant cell cycle control and mitotic checkpoint dysfunction.

### Correlation between LINC02657 expression and immune infiltration patterns in ccRCC

**Figure 5:**
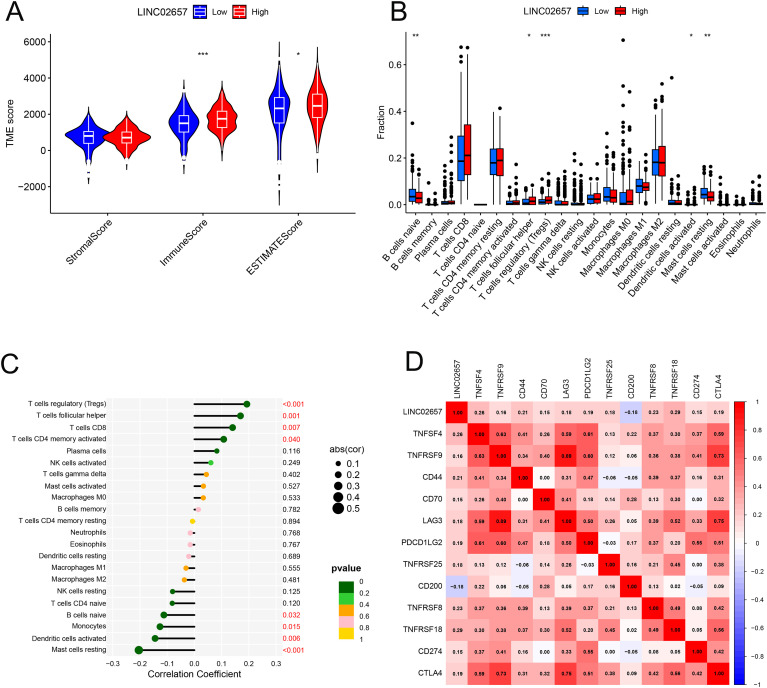
Correlation between LINC02657 expression and the tumor immune microenvironment in ccRCC. **(A)** Comparison of StromalScore, ImmuneScore, and ESTIMATEScore between high and low LINC02657 expression groups. **(B)** Distribution of 22 immune cell subtypes stratified by LINC02657 expression, as estimated by the CIBERSORT algorithm. **(C)** Spearman’s rank correlation analysis between LINC02657 expression and infiltrating immune cell subsets. **(D)** Pearson correlation analysis between LINC02657 expression and representative immune checkpoint molecules. For correlation analyses, two-sided P values < 0.05 were considered statistically significant. * indicates P < 0.05, ** indicates P < 0.01, and *** indicates P < 0.001.

**Figure 5A** presents a comparative analysis of tumor microenvironment (TME) scores, including StromalScore, ImmuneScore, and ESTIMATEScore, between high and low LINC02657 expression groups. Tumors with high LINC02657 expression exhibited significantly elevated ImmuneScore and ESTIMATEScore, indicating a higher proportion of immune and stromal components within the tumor microenvironment. **Figure 5B** illustrates the relative abundance of 22 immune cell subtypes across LINC02657 expression groups, revealing distinct immune infiltration patterns associated with LINC02657 expression levels. **Figure 5C** further demonstrates significant correlations between LINC02657 expression and specific immune cell populations. Positive correlations were observed with CD4+ memory T cells, CD8+ T cells, follicular helper T cells, and regulatory T cells (Tregs), whereas negative correlations were identified with resting mast cells, activated dendritic cells, monocytes, and naïve B cells. **Figure 5D** shows Pearson correlation analyses between LINC02657 expression and representative immune checkpoint-related molecules, including TNFSF4, TNFRSF18, and CD44, all of which exhibited significant positive associations. Importantly, these analyses indicate that LINC02657 expression is closely associated with immune cell infiltration patterns and immune checkpoint-related gene expression within the ccRCC tumor microenvironment. Collectively, these results demonstrate robust correlations between LINC02657 expression and immune-related features of the tumor microenvironment. However, these observations are based on transcriptomic association analyses and do not establish a direct causal role for LINC02657 in immune regulation.

### Drug sensitivity analysis and its clinical implications

**Figure 6:**
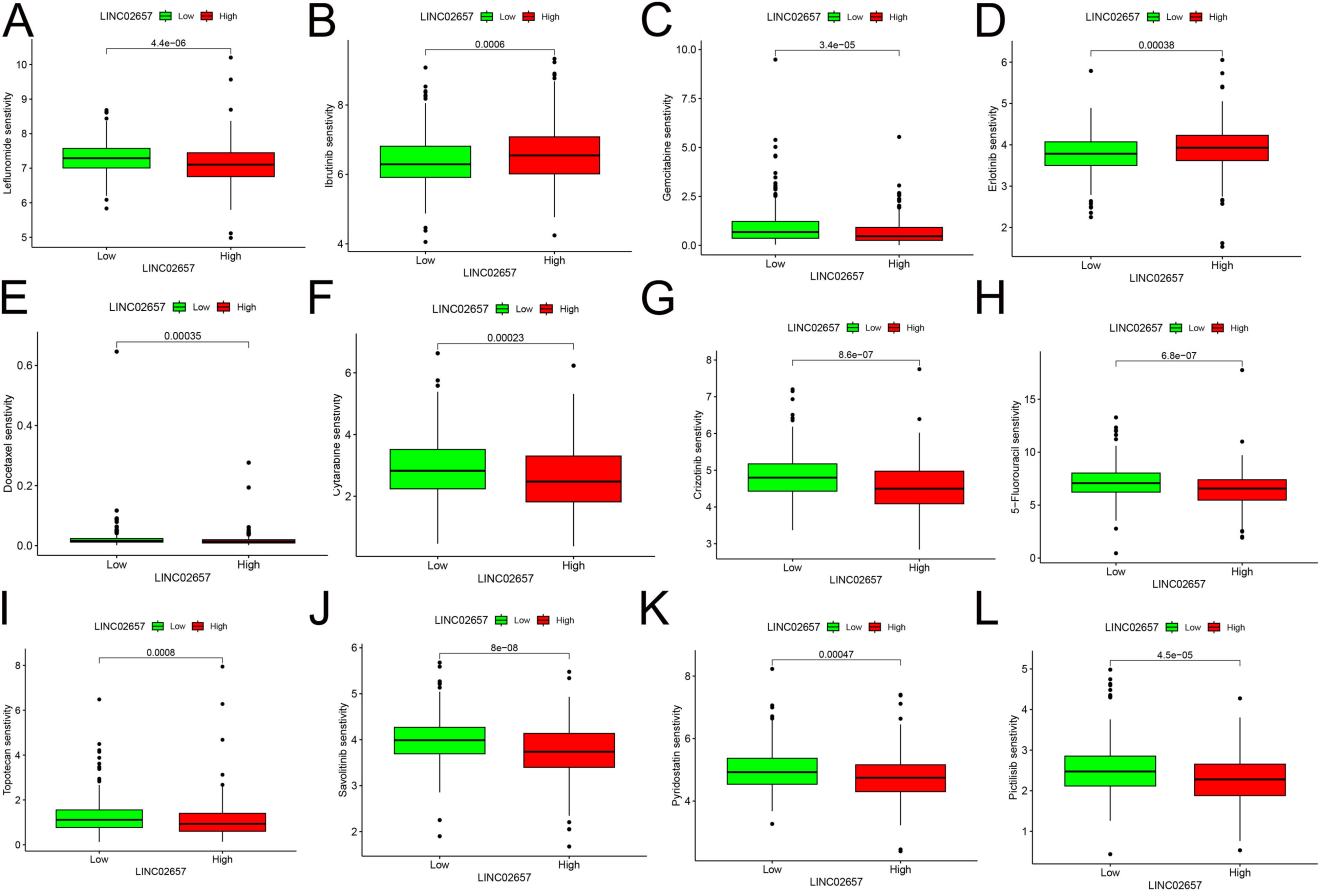
Correlation between LINC02657 expression and drug sensitivity in ccRCC. **(A–L)** Drug sensitivity analysis based on GDSC2 database predictions,.

Drug sensitivity predictions from the GDSC2 database (**Figures 6A-L**)revealed that the low-expression group of LINC02657 demonstrated significantly higher sensitivity to several chemotherapeutic agents, including docetaxel, gemcitabine, and 5-fluorouracil, as well as the targeted therapy Savolitinib. These associations were derived from GDSC-based in silico modeling and indicate a correlation between LINC02657 expression and predicted drug response rather than direct evidence of functional modulation of pharmacologic sensitivity. Accordingly, these results suggest that LINC02657 may serve as a potential predictive biomarker for drug response at the computational level, providing a hypothesis-generating framework for future experimental validation.

### Differential expression of LINC02657 in renal cancer cell lines and validation of knockdown efficiency

**Figure 7:**
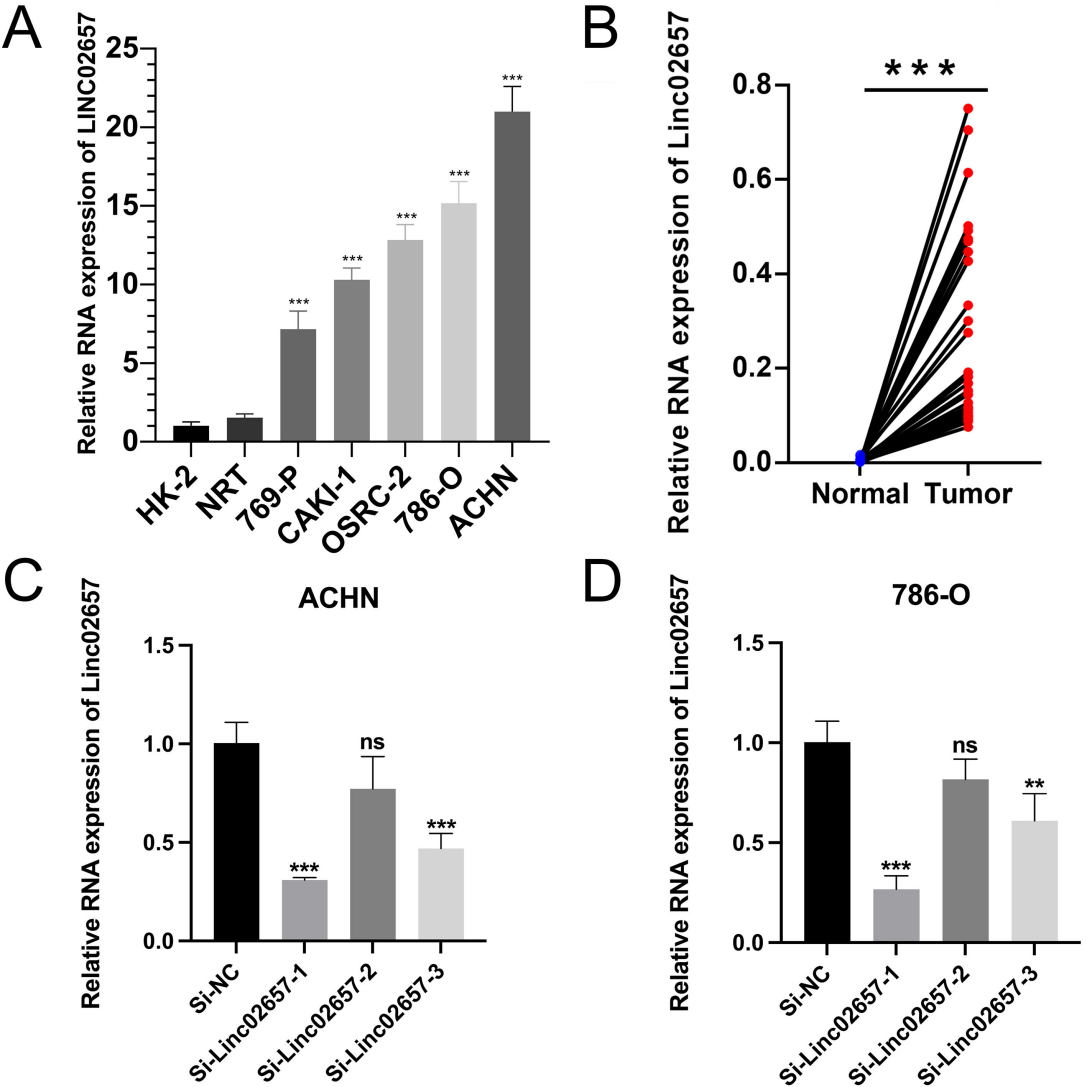
Expression profiling and knockdown validation of LINC02657 in renal cancer cell lines. **(A)** Quantitative analysis of LINC02657 expression levels in renal cancer cell lines (786-O, 769-P, ACHN, CAKI-1) compared to normal renal proximal tubular epithelial cells (HK-2) and normal renal tissues (NRT). **(B)** Expression of LINC02657 in human renal cell carcinoma tissues and paired adjacent non-tumor tissues, as determined by quantitative PCR (qPCR). **(C)** Knockdown efficiency of LINC02657 in ACHN cells transfected with three independent siRNAs (si-LINC02657-1, si-LINC02657-2, si-LINC02657-3) or negative control siRNA (si-NC). Data are presented as mean ± SD.**(D)** Knockdown efficiency of LINC02657 in 786-O cells transfected with three independent siRNAs (si-LINC02657-1, si-LINC02657-2, si-LINC02657-3) or negative control siRNA (si-NC). Data are presented as mean ± SD. (P < 0.05, *P < 0.01, **P < 0.001; NS, not significant).

As shown in **Figure 7A**, the expression levels of LINC02657 were significantly upregulated in renal cancer cell lines, including 786-O, 769-P, ACHN, and CAKI-1, compared with normal renal proximal tubular epithelial cells (HK-2) and normal renal tissues (NRT), indicating aberrant overexpression of LINC02657 in renal cancer cells (*P < 0.05, **P < 0.01, ***P < 0.001). Consistently, quantitative PCR analysis demonstrated that LINC02657 expression was markedly higher in human renal cell carcinoma tissues than in paired adjacent non-tumorous tissues (**Figure 7B**), further supporting its potential oncogenic role in renal cell carcinoma. To validate knockdown efficiency, three independent siRNAs targeting LINC02657 were transfected into 786-O and ACHN cells. As illustrated in **Figure 7C**, si-LINC02657–1 and si-LINC02657–3 significantly reduced LINC02657 expression, whereas si-LINC02657–2 did not achieve a statistically significant knockdown compared with the negative control (si-NC). si-LINC02657-1, which showed the highest knockdown efficiency, was used in subsequent experiments.

### Inhibition of cell proliferation and cell cycle progression by LINC02657 knockdown in renal cancer cell lines

**Figure 8:**
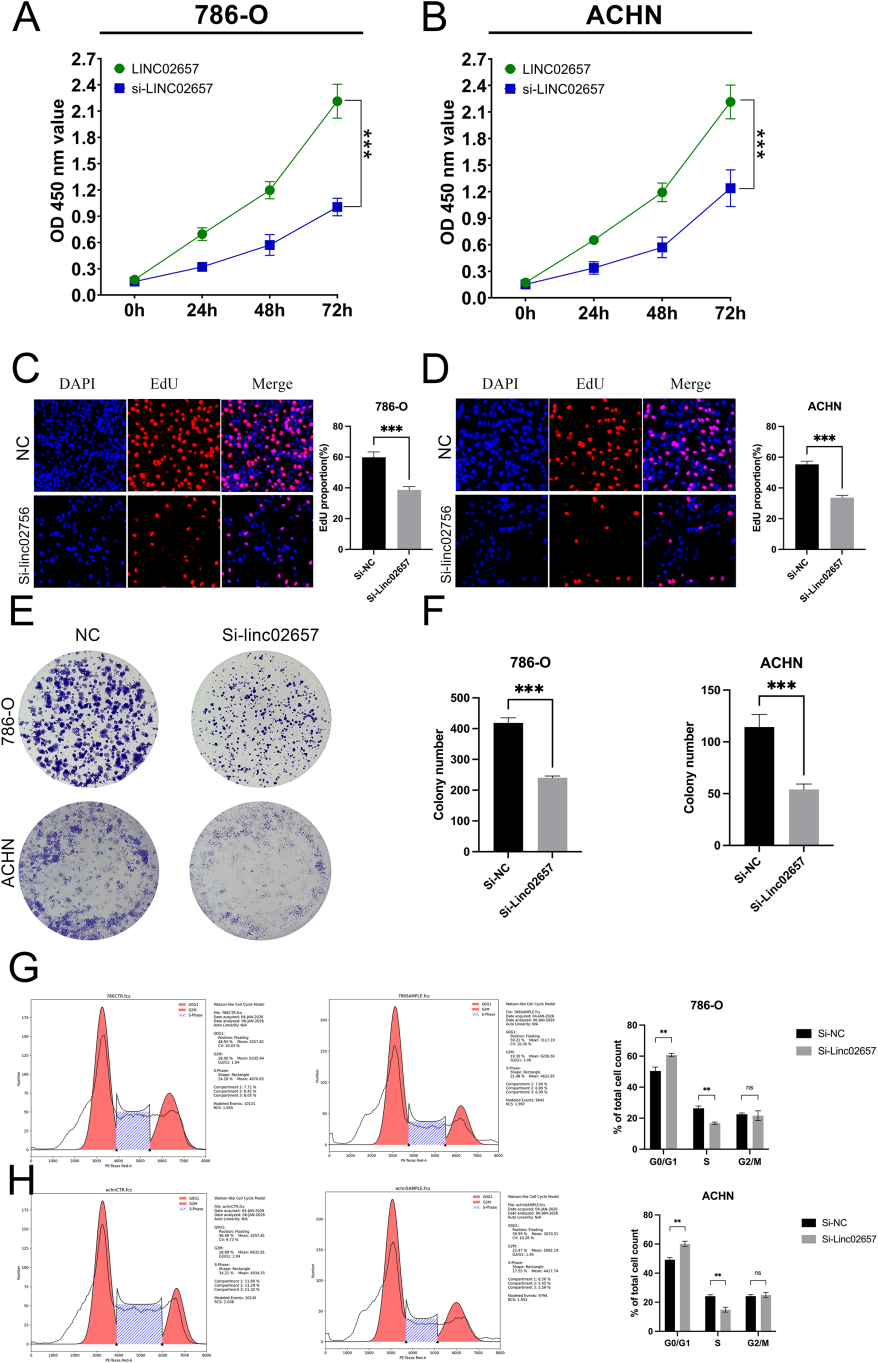
Knockdown of LINC02657 inhibits proliferation and colony formation, and induces cell cycle arrest in renal cancer cells. **(A, B)** Proliferation of 786-O **(A)** and ACHN **(B)** cells transfected with si-LINC02657 or negative control siRNA (si-NC), as measured by CCK-8 assay at indicated time points. **(C, D)** Representative images (left) and quantitative analysis (right) of EdU incorporation assays in 786-O **(C)** and ACHN **(D)** cells after LINC02657 knockdown. EdU-positive cells (proliferating cells) are shown in red, and nuclei (DAPI) are shown in blue. **(E, F)** Colony formation capability of 786-O and ACHN cells upon LINC02657 knockdown. **(E)** Representative images of crystal violet-stained colonies. **(F)** Quantitative analysis of the number of colonies formed. **(G, H)** Cell cycle distribution analysis of 786-O **(G)** and ACHN **(H)** cells after LINC02657 knockdown. The left panels show representative flow cytometry histograms, and the right panels show the quantitative percentages of cells in G0/G1, S, and G2/M phases. (*P < 0.05, **P < 0.01, ***P < 0.001).

As shown in **Figures 8A, B**, CCK-8 assays demonstrated that knockdown of LINC02657 significantly suppressed the proliferation of 786-O and ACHN cells compared with the negative control group (si-NC) at the indicated time points. Consistently, EdU incorporation assays revealed a marked decrease in the proportion of EdU-positive cells following LINC02657 silencing in both 786-O and ACHN cells (**Figures 8C, D**), indicating reduced DNA synthesis and proliferative activity. Furthermore, colony formation assays showed that LINC02657 knockdown significantly impaired the clonogenic ability of renal cancer cells. Both the number and size of colonies formed by 786-O and ACHN cells were markedly reduced upon LINC02657 silencing (**Figures 8E, F**). To further explore the underlying mechanism, cell cycle distribution was analyzed by flow cytometry. As illustrated in **Figures 8G, H**, depletion of LINC02657 resulted in a significant accumulation of cells in the G0/G1 phase, accompanied by a reduction in the S phase population in both cell lines, indicating that LINC02657 knockdown induces G0/G1 cell cycle arrest.

### Impact of LINC02657 knockdown on migration and invasion in renal cancer cell lines

**Figure 9:**
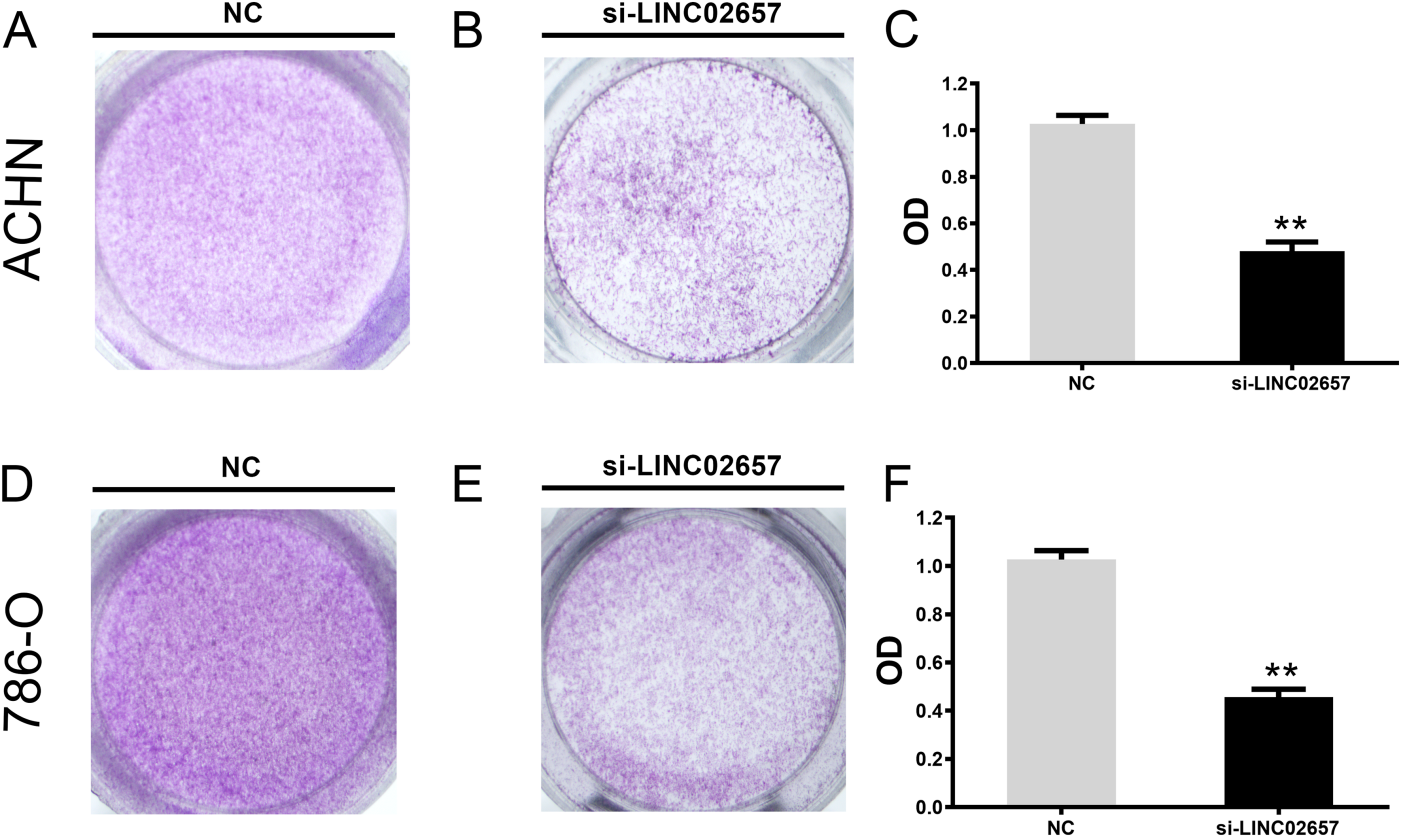
Knockdown of LINC02657 suppresses migration of renal cancer cells. **(A-C)** Migration of ACHN cells transfected with si-LINC02657 or negative control (si-NC), as determined by Transwell assay (uncoated membrane). **(D-F)** Migration of 786-O cells transfected with si-LINC02657 or si-NC, assessed by Transwell assay. Representative images (left) and quantification (right) are shown.

**Figure 10:**
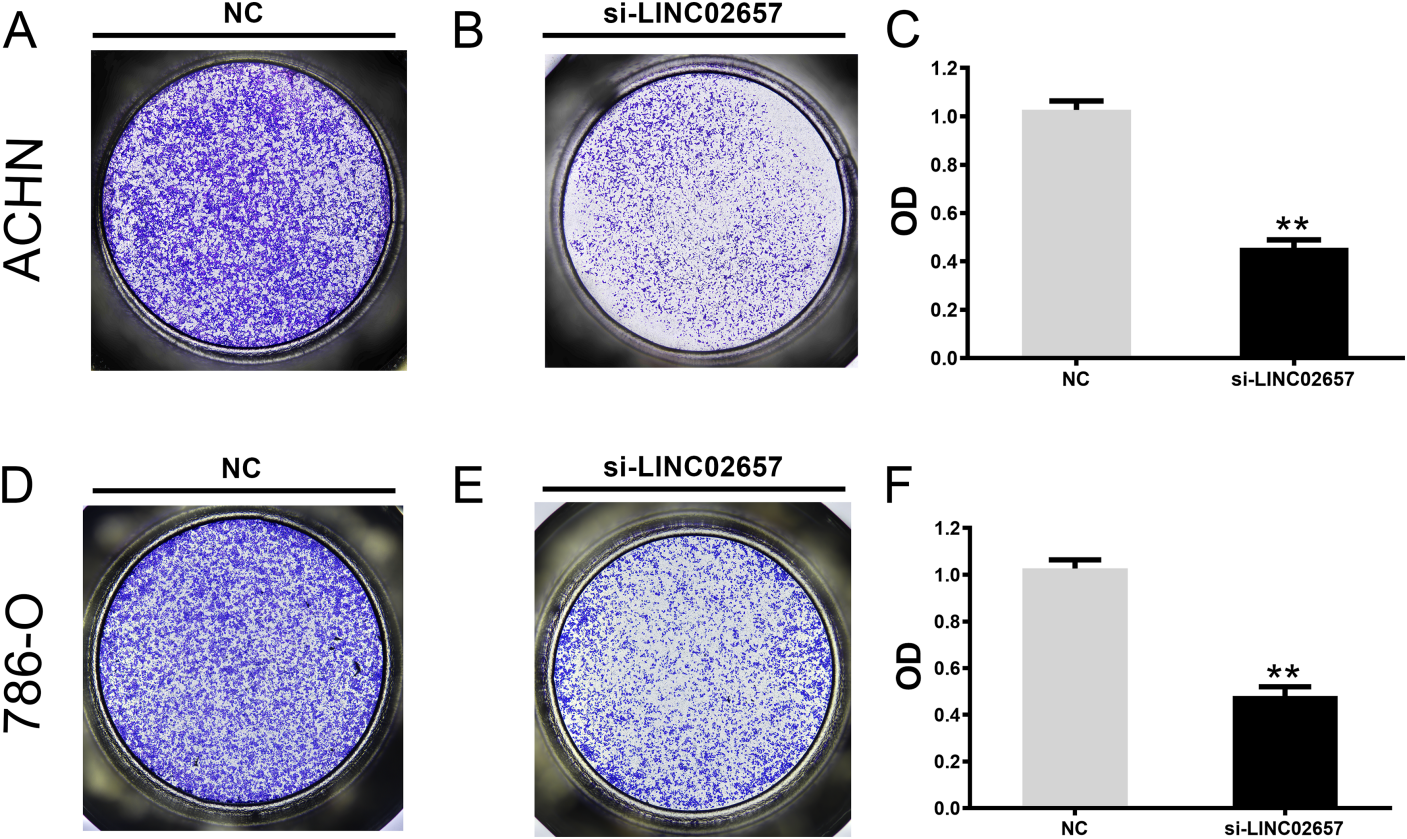
Knockdown of LINC02657 inhibits invasion of renal cancer cells. **(A-C)** Invasion of ACHN cells transfected with si-LINC02657 or si-NC, evaluated by Matrigel-coated Transwell assay. **(D-F)** Invasion of 786-O cells transfected with si-LINC02657 or si-NC, measured by Matrigel-coated Transwell assay. Representative images (left) and quantified results (right) are shown.

The influence of LINC02657 silencing on cell migration and invasion was assessed using Transwell assays in ACHN and 786-O renal carcinoma cell lines. As illustrated in **Figures 9A-F**, a significant reduction in cell migration was observed following LINC02657 knockdown, indicating a pronounced inhibition of migratory capacity in both cell lines. Similarly, **Figures 10A-F** demonstrate that the invasive ability of ACHN and 786-O cells was notably suppressed upon LINC02657 silencing. These findings provide strong evidence that LINC02657 is a critical regulator of cellular migration and invasion in renal cancer, and its knockdown impedes these essential cancer cell behaviors.

### Impact of LINC02657 knockdown on EMT-related protein expression

**Figure 11:**
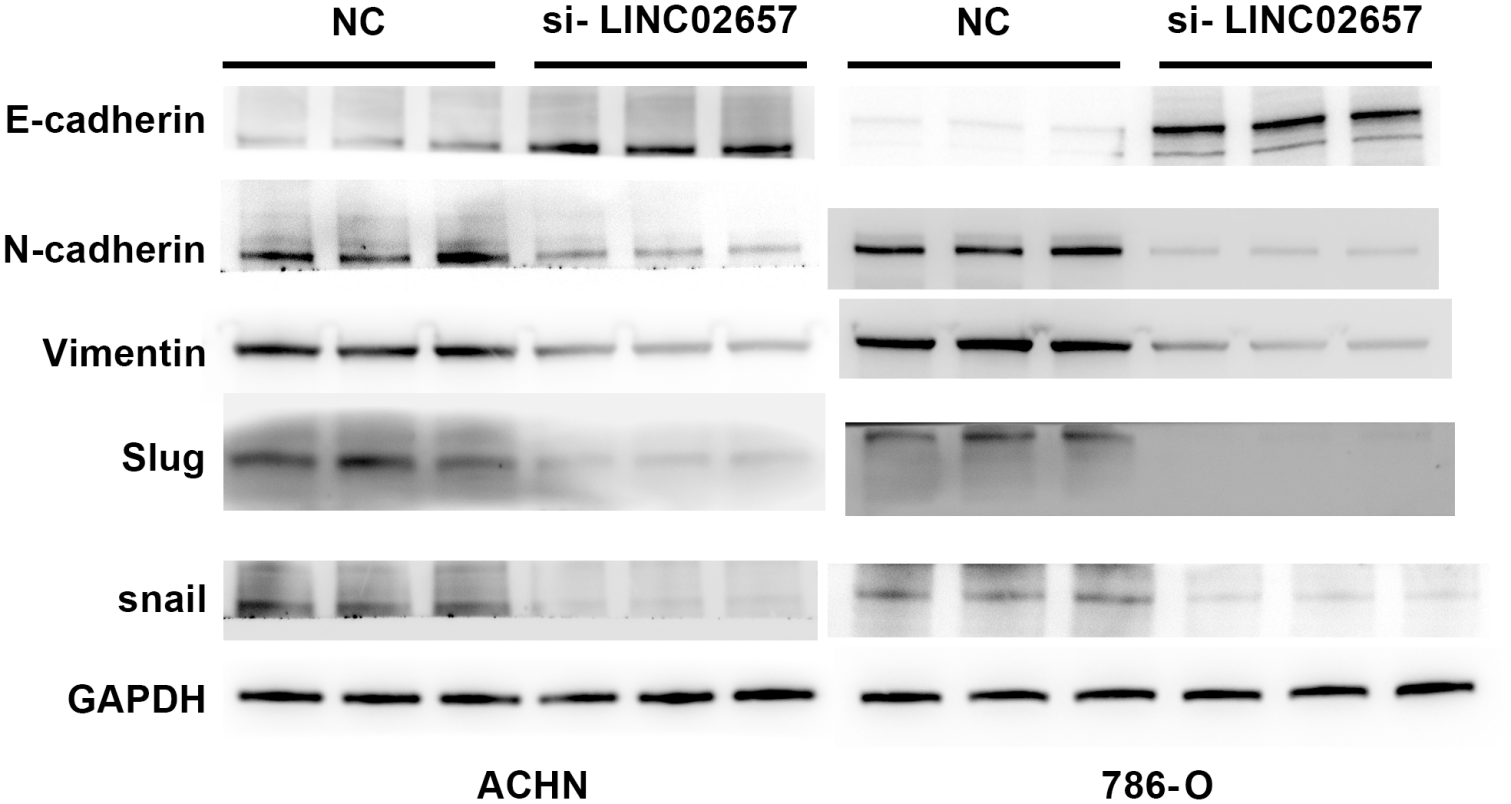
Knockdown of LINC02657 reverses epithelial-mesenchymal transition (EMT) in renal cancer cells. Western blot analysis of EMT-related markers in ACHN and 786-O cells transfected with si-LINC02657 or negative control (si-NC).

**Figure 11** presents the assessment of EMT-associated protein expression following LINC02657 knockdown in the ACHN and 786-O cell lines. The results demonstrate a significant increase in E-cadherin expression in the experimental groups compared to the control, indicating a shift towards epithelial phenotype. In contrast, a marked reduction in the levels of mesenchymal markers, including N-cadherin, vimentin, and Slug, was observed following LINC02657 silencing. These findings collectively indicate that LINC02657 knockdown suppresses epithelial–mesenchymal transition (EMT), as evidenced by coordinated changes in epithelial and mesenchymal markers, and is associated with reduced migratory and invasive potential.

## Discussion

The integrative bioinformatic and experimental analyses conducted in this study establish LINC02657 as a pivotal regulator in clear cell renal cell carcinoma (ccRCC), with profound implications for prognosis, tumor microenvironment (TME) modulation, and therapeutic response. Our findings demonstrate that LINC02657 is significantly upregulated in ccRCC tissues compared to normal renal tissues, a pattern consistent with its overexpression across multiple cancer types, as revealed by our pan-cancer analysis. This upregulation, coupled with its strong correlation with adverse clinicopathological features—such as advanced tumor grade, clinical stage, primary tumor size, and distant metastasis—positions LINC02657 as a promising biomarker for aggressive disease phenotypes in ccRCC. These results align with recent studies highlighting the oncogenic roles of long non-coding RNAs (lncRNAs) in renal cell carcinoma, where lncRNAs such as HOTAIR, MALAT1, and PVT1 have been implicated in tumor progression, metastasis, and epigenetic regulation (11, 15–20). The prognostic significance of LINC02657 is robustly supported by its association with reduced overall survival (OS), disease-specific survival (DSS), and progression-free survival (PFS) in the TCGA-KIRC cohort, with consistent trends validated in the BSET and ICGC-RECA datasets. Multivariate Cox regression analyses confirmed LINC02657 as an independent prognostic factor for OS (HR = 2.041, 95% CI: 1.536–2.713, P < 0.001), surpassing the predictive strength of tumor grade. However, its attenuated significance for PFS in multivariate models (HR = 1.061, P = 0.086) suggests that LINC02657’s prognostic impact may be modulated by traditional pathological factors, particularly in earlier disease stages. This context-dependent effect may reflect molecular heterogeneity or interactions with ccRCC-specific drivers, such as VHL mutations or HIF pathway dysregulation, which are prevalent in ccRCC (21–26). Further studies are needed to elucidate these interactions, particularly in diverse patient cohorts. Importantly, LINC02657 expression showed no significant association with patient age or gender, suggesting that its dysregulation in ccRCC is unlikely to be driven by demographic factors. Instead, its strong correlations with tumor grade, stage, and metastatic status indicate that LINC02657 expression more likely reflects tumor-intrinsic biological aggressiveness. Moreover, the cohort-specific prognostic inconsistency observed between the TCGA-KIRC and ICGC datasets may be attributable to differences in sample size, population composition, clinical annotation, follow-up duration, treatment heterogeneity, and data processing pipelines, underscoring the molecular heterogeneity of ccRCC and the importance of multi-cohort validation.

It is important to clarify the relationship between the present study and our previous work (6), in which LINC02657 was identified as one component of a programmed cell death–related lncRNA prognostic signature for ccRCC. That prior study was designed as a population-level, hypothesis-generating analysis aimed at risk stratification and therapeutic response prediction based on a multigene model, without functional or mechanistic investigation of individual lncRNAs. In contrast, the current study deliberately shifts the focus from prognostic modeling to gene-specific biological characterization. Here, LINC02657 was selected for in-depth analysis to determine whether it represents a functionally active driver of ccRCC progression rather than a passive marker within a composite risk score. By integrating transcriptomic analyses, tumor microenvironment profiling, drug sensitivity prediction, and *in vitro* functional assays, we provide mechanistic and experimental evidence supporting the oncogenic role of LINC02657 in ccRCC. Thus, the present findings biologically substantiate and extend our previous statistical observations, advancing LINC02657 from a risk model–associated lncRNA to a mechanistically and clinically relevant regulator in ccRCC.

Functionally, LINC02657 appears to drive oncogenesis through dysregulation of critical cellular processes, as evidenced by our differential gene expression and functional enrichment analyses. The enrichment of LINC02657-associated genes in pathways related to cell cycle progression, mitotic regulation, and p53 signaling suggests a role in promoting tumor cell proliferation and genomic instability. These findings are consistent with recent reports linking lncRNAs to cell cycle dysregulation and mitotic checkpoint dysfunction in cancer (27–29). The significant enrichment in metabolic pathways, including oxidative phosphorylation and retinol metabolism, implicates LINC02657 in metabolic reprogramming, a hallmark of ccRCC driven by HIF-mediated alterations (30, 31). Additionally, the enrichment in infection-related pathways (e.g., Helicobacter pylori and Vibrio cholerae signaling) may reflect cross-talk between inflammatory signaling and tumorigenesis, a phenomenon increasingly recognized in renal cancer (32). These pathways warrant further exploration to clarify their relevance to ccRCC pathogenesis. Notably, several enriched KEGG pathways were annotated as infection-related signatures. These pathways are unlikely to indicate true microbial involvement in ccRCC but instead reflect shared gene components associated with inflammatory signaling, cell adhesion, cytoskeletal remodeling, and stress-response processes that are frequently co-opted during tumor progression. In contrast, the enrichment of cancer-relevant pathways, including PI3K–AKT signaling, MAPK signaling, focal adhesion, and ECM–receptor interaction, is biologically plausible and consistent with established mechanisms underlying ccRCC invasion and metastasis. Accordingly, our interpretation focuses on these tumor-associated pathways rather than infection-specific annotations.

The tumor microenvironment analyses revealed significant associations between LINC02657 expression and the immune landscape of ccRCC. Elevated ImmuneScore and ESTIMATEScore in the high LINC02657 expression group, together with increased infiltration of CD4+ memory T cells, CD8+ T cells, follicular helper T cells, and regulatory T cells (Tregs), indicate that LINC02657 expression is correlated with T cell–enriched immune features, while also being associated with increased Treg presence. Importantly, increased immune cell infiltration does not necessarily reflect effective anti-tumor immunity. In ccRCC, elevated infiltration of CD8^+^ T cells may coexist with immune dysfunction or exhaustion, particularly in the context of increased regulatory T cell presence and immune checkpoint activation, thereby contributing to poorer clinical outcomes despite higher immune cell abundance. In addition, positive correlations were observed between LINC02657 expression and multiple immune checkpoint molecules, including TNFSF4, TNFRSF18, and CD44, suggesting a potential link between LINC02657 expression and immune checkpoint–related pathways. These observations are based on correlative analyses and do not establish a direct mechanistic role of LINC02657 in immune regulation. Nevertheless, the identified immune infiltration patterns and checkpoint associations are consistent with previous reports describing lncRNA-associated immune heterogeneity in ccRCC, in which lncRNAs are linked to variations in immune cell composition and checkpoint expression (6, 10, 33). The negative correlations with resting mast cells, activated dendritic cells, and monocytes further suggest that LINC02657 expression is associated with selective differences in innate immune cell infiltration. Collectively, these findings indicate that LINC02657 expression is linked to a complex immune microenvironment in ccRCC, which may have implications for immunotherapy response and warrants further functional investigation (34, 35).

The drug sensitivity analysis revealed that low LINC02657 expression is associated with increased sensitivity to chemotherapeutic agents (e.g., docetaxel, gemcitabine, 5-fluorouracil) and targeted therapies (e.g., Savolitinib), suggesting that LINC02657 expression is associated with differences in predicted therapeutic vulnerability based on computational modeling. This is particularly relevant given the limited efficacy of traditional chemotherapies in ccRCC and the growing reliance on targeted therapies and immunotherapies (36). The identification of LINC02657 as a potential biomarker for drug response underscores its clinical utility in personalizing treatment strategies, a cornerstone of precision oncology (37).

Our *in vitro* experiments further validated the oncogenic role of LINC02657 in ccRCC. Knockdown of LINC02657 in 786-O and ACHN cell lines significantly inhibited cell proliferation, migration, and invasion, consistent with its role in promoting aggressive tumor phenotypes. The suppression of epithelial-to-mesenchymal transition (EMT) markers, including reduced N-cadherin, vimentin, and Slug expression, alongside increased E-cadherin, suggests that LINC02657 contributes to metastatic potential, at least in part, through modulation of EMT-related processes. These findings are in line with recent studies linking lncRNAs to EMT in ccRCC, where lncRNAs act as molecular sponges for miRNAs targeting EMT-related genes (38, 39).

Despite these insights, several limitations must be acknowledged. The retrospective nature of the TCGA and ICGC datasets may introduce selection bias, and the lack of significant prognostic associations in the ICGC cohort for OS suggests cohort-specific differences that require further investigation. Additionally, while our *in vitro* experiments confirm the functional role of LINC02657, *in vivo* studies are needed to validate these findings in a physiological context. The molecular mechanisms underlying LINC02657’s regulation of immune infiltration and drug sensitivity, potentially through interactions with miRNAs or transcription factors, also warrant further exploration. In addition, functional validation was conducted in two representative ccRCC cell lines, and further studies incorporating additional cell models and patient-derived samples are warranted to enhance the generalizability and translational relevance of these findings. Additionally, the drug sensitivity analysis was based on GDSC-derived computational predictions rather than direct pharmacological experiments, which limits causal interpretation of therapeutic response.

In conclusion, LINC02657 emerges as a multifaceted regulator in ccRCC, influencing tumor progression, immune modulation, and therapeutic response. Its robust prognostic and diagnostic potential, combined with its functional role in promoting proliferation, metastasis, and EMT, positions LINC02657 as a promising biomarker and therapeutic target. These findings pave the way for future mechanistic studies and clinical trials to explore LINC02657-targeted interventions in ccRCC, potentially improving patient outcomes in this challenging malignancy.

## Data Availability

The original contributions presented in the study are included in the article/**Supplementary Material**. Further inquiries can be directed to the corresponding author/s.

## Supplementary material

Supplementary Figure 1 Supplementary analyses of the prognostic value and clinical associations of LINC02657. **(A, B)** Forest plots showing no significant association was found between LINC02657 expression and overall survival in the disease-free survival in the TCGA cohort (DFS-TCGA) **(A)** or ICGC cohort (OS-ICGC) **(B, C)** Time-dependent receiver operating characteristic (ROC) curves ana-lyzing the predictive accuracy of LINC02657 expression for 1-year, 3-year, and 5-year overall sur-vival in the TCGA-KIRC cohort. The area under the curve (AUC) values for each time point are indicated. **(D, E)** Box plots demonstrating that LINC02657 expression levels are not significantly associated with patient age **(D)** or gender **(E)** in the TCGA-KIRC cohort.
