# Supplementary figures and images for: Unraveling the role of LINC02657 in clear cell renal cell carcinoma: insights into tumor aggression, immune modulation, and treatment response
Source: Front Immunol. 2026 Feb 17;17:1735169. doi: 10.3389/fimmu.2026.1735169 (PMC12953511; doi:10.3389/fimmu.2026.1735169)

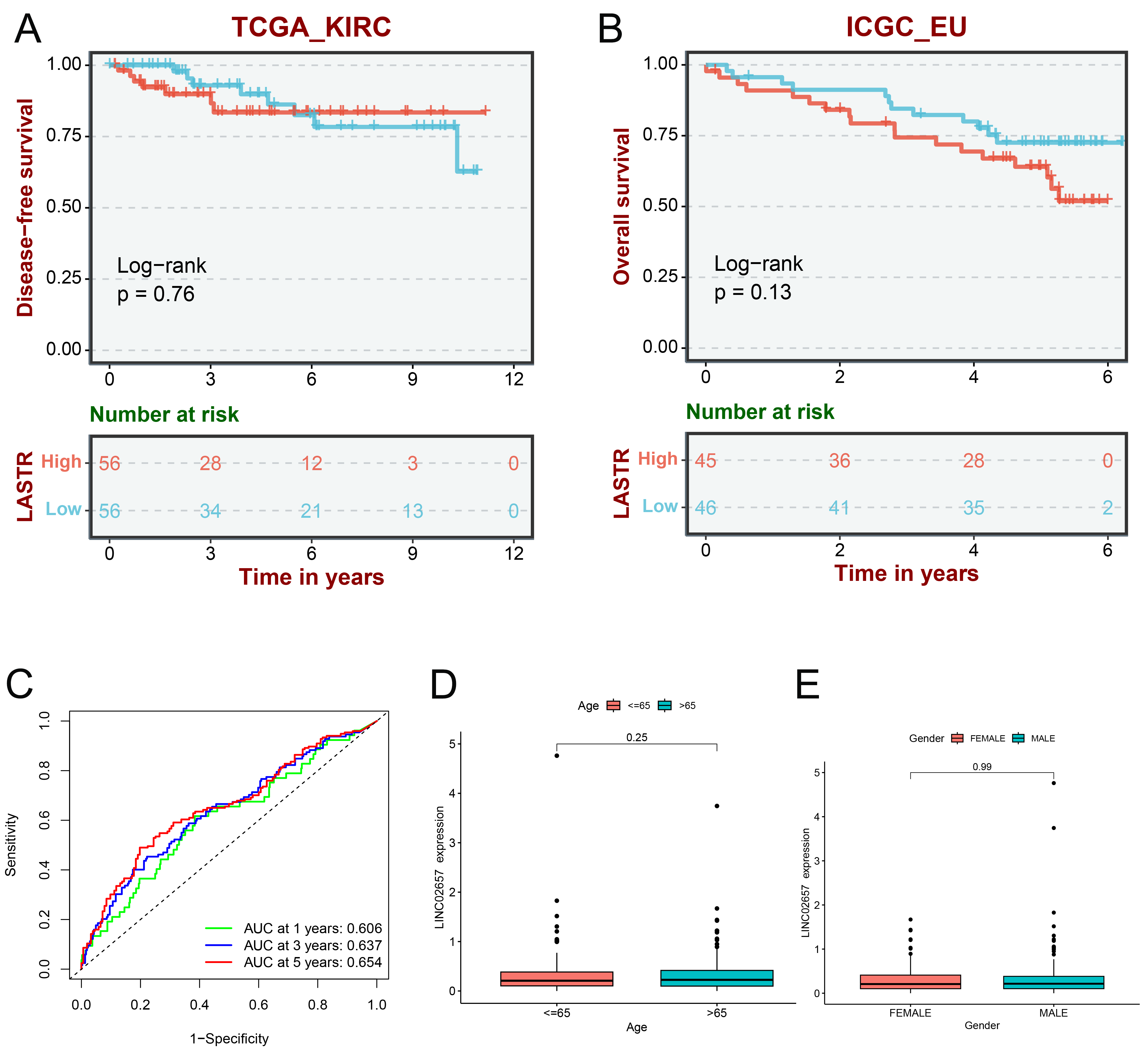

Supplement: Supplementary Figure 1 — Supplementary analyses of the prognostic value and clinical associations of LINC02657. (A, B) Forest plots showing no significant association was found between LINC02657 expression and overall survival in the disease-free survival in the TCGA cohort (DFS-TCGA) (A) or ICGC cohort (OS-ICGC) (B, C) Time-dependent receiver operating characteristic (ROC) curves ana-lyzing the predictive accuracy of LINC02657 expression for 1-year, 3-year, and 5-year overall sur-vival in the TCGA-KIRC cohort. The area under the curve (AUC) values for each time point are indicated. (D, E) Box plots demonstrating that LINC02657 expression levels are not significantly associated with patient age (D) or gender (E) in the TCGA-KIRC cohort. [file Image1.tif]
