# Supplementary material for: Unraveling the role of LINC02657 in clear cell renal cell carcinoma: insights into tumor aggression, immune modulation, and treatment response
Source: Front Immunol. 2026 Feb 17;17:1735169. doi: 10.3389/fimmu.2026.1735169 (PMC12953511; doi:10.3389/fimmu.2026.1735169)
Supplement: Supplementary file 2 [file Table1.docx]

**Supplementary Table S1. Oligonucleotide sequences used for quantitative PCR and RNA interference**

| **Production No.** | **Name** | **Sequence (5′ → 3′)** | **Length (nt)** |
| --- | --- | --- | --- |
| 2809719 | LINC02657-F | GGAGTTGAATTGTCTCTGTAG | 21 |
| 2809720 | LINC02657-R | TATTGCTTGGATGTGAGTCT | 20 |
| 2779051 | hLINC02657 si-1-sense | GACUCACAUCCAAGCAAUAAATT | 23 |
| 2779052 | hLINC02657 si-1-antisense | UUUAUUGCUUGGAUGUGAGUCTT | 23 |
| 2779053 | hLINC02657 si-2-sense | CACAGGGUUAAUGACUCAAUUTT | 23 |
| 2779054 | hLINC02657 si-2-antisense | AAUUGAGUCAUUAACCCUGUGTT | 23 |
| 2779055 | hLINC02657 si-3-sense | GGGAAAUUCAGAUCAUCUAAATT | 23 |
| 2779056 | hLINC02657 si-3-antisense | UUUAGAUGAUCUGAAUUUCCCTT | 23 |
